# Seeking lifestyle counselling at primary health care centres: a cross-sectional study in the Swedish population

**DOI:** 10.1186/s12875-023-02035-3

**Published:** 2023-03-20

**Authors:** Frida Lundin Gurné, Per-Arne Svensson, Ida Björkman, Eva Lidén, Sofie Jakobsson

**Affiliations:** 1 Institute of Health and Care Sciences, The Sahlgrenska Academy, University of Gothenburg, 40530 Gothenburg, SE Sweden; 2 Centre for Person-Centred Care (GPCC), University of Gothenburg, Gothenburg, Sweden

**Keywords:** Cross-sectional study, Health behaviour, Lifestyle counselling, Lifestyles, Primary health care, Primary health care centre

## Abstract

**Background:**

Millions of people follow an unhealthy lifestyle in terms of tobacco consumption, hazardous use of alcohol, poor eating habits, and insufficient physical activity. Healthy lifestyles can to a large extent prevent and/or delay progression of non-communicable diseases. Factors influencing persons health-seeking behaviour regarding unhealthy lifestyles are of importance for sustainable health-promotive and disease-preventive work in primary health care. Generally, lifestyle interventions within primary health care are seen as feasible, but rarely reach all members of the general population. Few studies have been conducted about the likelihood among the general population to voluntarily contact a primary health care centre for support regarding lifestyle changes. The present study therefore aimed to investigate the general population’s likelihood of contacting a primary health care centre regarding their lifestyles, and factors associated with a lower such likelihood.

**Methods:**

A probability sample of adults living in Sweden (*n* = 3 750) were invited to participate in a cross-sectional survey regarding how societal developments affect attitudes and behaviours of the adult Swedish population. Data were collected between September and December 2020. Participants completed a questionnaire about lifestyle changes, and the data were analysed using descriptive statistics, Chi-square test and logistic regression analysis.

**Results:**

The response rate was 52.0% (*n* = 1 896). Few persons responded that they would be likely to contact a primary health care centre for support regarding their lifestyles. Factors predicting a lower likelihood of contacting primary health care included few yearly visits to a primary health care centre, male sex, and living in a rural area.

**Conclusions:**

Primary health care centres are not the first choice for lifestyle counselling for the majority of adults living in Sweden. We have identified factors predicting low likelihood of using the support available at these centres. In order to work with sustainable and visible health-promotive and disease-preventive strategies at primary health care centres, these settings need to find valid methods to involve and collaborate with the members of the general community, to meet the needs of a population struggling with unhealthy lifestyles.

## Background

Lifestyle risk factors such as tobacco consumption, high consumption of alcohol, unhealthy eating habits, and insufficient physical activity lead to major global health problems. These risk factors make a large contribution to non-communicable diseases (NCDs) such as cardiovascular diseases, cancer, chronic respiratory diseases, and type 2 diabetes [1]. Healthy lifestyles can to a large extent prevent NCDs and/or delay progression of NCDs [1, 2], promote self-assessed general health [1], and reduce the burden on the health care budget [3]. Sweden, like many other Western countries, is facing increasing rates of NCDs [4], in which approximately one-fifth of the overall care burden can be attributed to unhealthy lifestyles [5].

Offering specific lifestyle interventions for members of the general community can contribute to improved lifestyles in a population [6, 7]. In a Swedish study Brobeck et al. [8] reported that approximately 40% of the patients changed their lifestyles when health care professionals working in primary health care addressed lifestyle changes with supplementary advice in patient encounters, however with an uncertainty about the long-lasting effects. Furthermore, the EUROPREVIEW showed that most healthy patients without any diagnosis of lifestyle diseases such as cardiovascular diseases or type 2 diabetes wanted to visit health care professionals once a year or more often to check for example their blood pressure [9]. Primary health care has been proposed to be well placed to provide lifestyle counselling aimed at supporting the general population to adopt healthier lifestyles [10, 11]. However, such interventions may not reach the people in most need if they have not yet developed chronic diseases, or if they do not attend such clinical care settings [12]. Health-seeking behaviour within primary health care is influenced by several factors. For example, persons with low health literacy can experience greater difficulties in finding the ‘right’ care pathway [13, 14]. Smoking, regular alcohol consumption and physical inactivity are other factors that can decrease the probability of seeking primary health care [12]. Additionally, persons with risk factors for, for example cardiovascular diseases and/or obesity are more incline to seek care for weight loss in comparison with persons having overweight without risk factors for cardiovascular diseases [15]. Furthermore, a study published in 2018 showed that only a minority of persons with obesity sought help for weight loss the past year. Instead, they preferred self-guided efforts to manage ill health [16]. Another study reported that approximately 10% of overweight/obese adults sought care from either dieticians/nutritionist, personal trainer or doctors to lose weight [17]. However, in contrast, persons with obesity or overweight have reported a need of more help with weight management from physicians in primary health care [18]. Two population-based studies among British citizens showed that being a smoker was associated with reduced likelihood of seeking help for symptoms linked to their unhealthy lifestyle habit such as cough [19, 20], also the complex phenomena of lung cancer stigma delayed the intension to seek help [20].

In Sweden, several initiatives have been launched towards health-promotive and disease-preventive practices such as national target goals for public health in 2003 [21]. Additional plans for action were in 2011 and 2018 when national clinical guidelines for unhealthy lifestyles were released. These guidelines particularly addressed that health care professionals should support the general population to change tobacco- and alcohol consumption, to change eating habits and to increase levels of physical activity [5, 22]. There are more than 1100 primary health care centres available where different health care professionals work such as general practitioners, registered nurses, district nurses, physiotherapists, and psychologists. Since 2010, each member in the general population is free to select a primary health care provider of their choice [23]. It is of importance to address to which extent members of the general population seek community care for their lifestyles in order to support lifestyle changes among members of the general population. This article presents data from a Swedish survey conducted among persons aged between 16 and 85 years. The aim was to investigate the general population’s likelihood of contacting a primary health care centre regarding their lifestyles, and factors associated with a lower such likelihood.

## Methods

### Study design and participants

A cross-sectional survey was conducted with a nationally representative sample of persons living in Sweden. This was part of a large national survey conducted by the SOM Institute (society, opinion and media) at the University of Gothenburg, which aimed to investigate how societal developments affect the attitudes and behaviours of the adult Swedish population [24]. The total sample consisted of 3 750 invited participants aged between 16 and 85 years. Potential participants were identified through a probability sample provided by the Swedish Tax Authority from a pool which included all persons residing in Sweden at the end of August 2020.

### Data collection

The survey was sent to the participants’ home addresses by standard mail between September and December 2020, and reminders were sent by mail and text messages. The survey could be completed either by answering a paper-based questionnaire or by logging on to a digital platform using a code. The data collection period was 98 days, and the selected respondents could withdraw their participation at any time during this period. A note of thanks containing a lottery ticket to the value of 50 SEK (≈ 5 EUR) was sent to the participants who returned a completed questionnaire.

### Questionnaire

Likelihood of contacting a primary health care centre for support for lifestyle changes was assessed using a 4-point Likert scale regarding each of the four lifestyles investigated in this study: smoking habits, alcohol consumption, eating habits, and physical activity. The question was: “How likely is it that you would contact a primary health care centre if you needed support to change any of the following lifestyles?” with response options; “very likely”, “fairly likely”, “not likely”, and “not at all likely”. Number of visits to a primary health care centre during the last year was measured on a 5-point scale, with answer alternatives “none”, “one or two”, “three or four”, “five or six” and “seven or eight visits or more”. The responses to this question were stratified into “none”, “one or two”, “three or four”, and “five or more”. Participants were asked to self-report both their health and their perceived need to change any of the four lifestyles. Self-reported health was measured using a scale ranging from 0 to 10, with higher scores indicating better health, and stratified in the analysis into 0–4 = poor, 5–6 = fairly good, 7–8 = good, and 9–10 = very good health. To report need to change any of the four lifestyles, the participants could answer “yes” or “no” for smoking habits, alcohol consumption, eating habits, and physical activity, respectively.

Participants’ self-reported sex, age, education level, living area, and total household income were also used. Responses regarding age were stratified into four groups: 16–29 years, 30–49 years, 50–64 years, and 65–85 years. Participants reporting total household income of < 300 000 SEK per year were classified as low-income, those reporting 300 000–700 000 SEK were classified as middle-income, and those reporting > 700 000 SEK were classified as high-income.

### Statistical analysis

Descriptive statistics were used to describe sociodemographic characteristics and health-related factors. Likelihood of contacting a primary health care centre was stratified into “likely” (comprising the response alternatives “very likely” and “fairly likely”) and “not likely” (comprising the response alternatives “not likely” and “not at all likely”). The probability of answering likely to seek support from primary health care for lifestyle counselling was analysed in subgroups of perceived need to change that specific lifestyle habit using Chi-square test. Logistic regression was used to calculate the odds ratio (OR) for having lower likelihood of contacting a primary health care centre regarding lifestyle changes, with certain sociodemographic characteristics and health-related factors as predictors.

*P*-values < 0.05 were considered statistically significant, and no correction for multiple testing was performed. All data analyses were performed using version 27.0 of IBM SPSS Statistics (IBM Corp., Armonk, NY, USA). No missing values were imputed for the analysis. For the main question about likelihood of contacting a primary health care centre regarding lifestyle changes, some respondents did not answer that question, thus some data were missing, namely 99, 93, 69, and 80 persons respectively, for the four lifestyles (smoking habits, alcohol consumption, eating habits, and physical activity).

## Results

Of the 3 750 questionnaires that were distributed, 1 896 valid responses were returned, giving a response rate of 52.0%. Sociodemographic characteristics and health-related factors are presented in Table 1. Among the 1 896 participants, 1 837 answered the question about sex; 48.0% were men and 52.0% were women. The most common educational level ( 34.2%) was a post-secondary education of more than 3 years, the most common place of residence (48.9%) was a city (but not a “major city”; see Table 1), and the most common total household income bracket was middle-income (42.4%). Nearly half of the participants rated their self-reported health as good (46.5%). The most common reported frequency of visits to a primary health care centre in the past years was one or two (41.5%). Of the four lifestyles, physical activity was the one most commonly identified by the participants as in need of change (54.7%).

**Table 1:** Sociodemographic characteristics and health-related factors among the sample

Variable	Total (*n* = 1 896)	
	n	%
**Sex**		
Men	882	48.0
Women	955	52.0
Missing^a^	59	
**Age (years)**		
16–29	289	15.2
30–49	548	28.9
50–64	466	24.6
65–85	593	31.3
Missing^a^	0	
**Education**		
Primary education/school	265	14.5
Upper secondary education	530	29.0
Post-secondary education of < 3 years	407	22.3
Post-secondary education of > 3 years	626	34.2
Missing^a^	68	
**Living area**		
Major city^b^	338	18.4
City	898	48.9
Town	344	18.7
Rural area	257	14.0
Missing^a^	59	
**Total household income**^**c**^		
Low	390	22.4
Middle	737	42.4
High	613	35.2
Missing^a^	156	
**Self-reported health**		
Poor	151	8.2
Fairly good	267	14.5
Good	856	46.5
Very good	568	30.8
Missing^a^	54	
**Number of visits to a primary health care centre in the past year**		
None	379	20.5
One or two	769	41.5
Three or four	396	21.4
Five or more	309	16.7
Missing^a^	43	
**Need to change lifestyles**^**d**^		
Smoking habits	139	7.7
Alcohol consumption	215	11.9
Eating habits	800	43.7
Physical activity	1002	54.7

^a^ No / unclear answers

^b^Stockholm, Gothenburg, Malmö

^c^ Low income = up to 300 000 SEK; middle income = 300 000–700 000 SEK; high income =  > 700 000 SEK (1 SEK ≈ 0.10 EUR on 22 November 2021). ^d^ Participants may have given several response alternatives

**Fig. 1:**
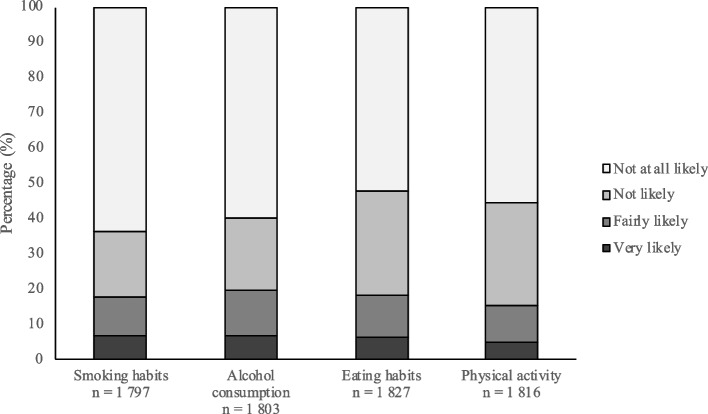
Likelihood of contacting a primary health care centre regarding support for lifestyle changes among all persons included. Values are given in %

Figure 1 shows the reported likelihood of contacting a primary health care centre regarding lifestyle changes. For all four lifestyles, “not at all likely” was the most common answer, ranging from 52.5% to 63.9%. When the responses were stratified as described earlier, the proportions of participants stating that they were “not likely” to contact a primary health care centre were 82.4% for smoking habits, 80.7% for alcohol consumption, 82.1% for eating habits and 84.9% for physical activity. In comparison between all four lifestyles, alcohol consumption had the highest percentage of participants who reported that they were “very likely” to seek support at a primary health care centre (6.6%).

**Fig. 2:**
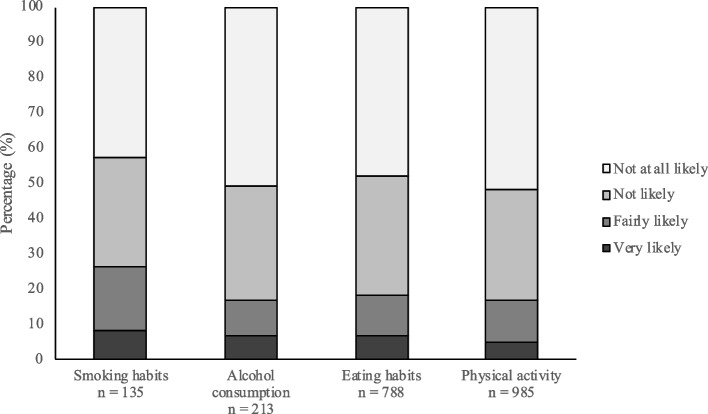
Likelihood of contacting a primary health care centre regarding support for lifestyle changes among participants that perceived a need to change each lifestyle habit. Values are given in %

Figure 2 shows participants who reported a need to change respective lifestyle habit in relation to the 4-point Likert scale of likelihood. For all four lifestyles, “not at all likely” was the most common answer, ranging from 43.0% to 51.8%. When the responses were stratified, smoking habits hade the highest percentages of all four lifestyles of participants who reported that they were “likely” to seek support at a primary health care centre (25.9%). Furthermore, Table 2 shows the stratified response alternatives divided by sociodemographic characteristics and health-related factors.

**Table 2:** Likelihood of contacting a primary health care centre for lifestyle changes

	**Smoking habits**		**Alcohol consumption**		**Eating habits**		**Physical activity**	
**Participants (n)**	*n* = 1 797		*n* = 1 803		*n* = 1 827		*n* = 1 816	
**Likelihood (%)**	Likely	Not likely	Likely	Not likely	Likely	Not likely	Likely	Not likely
**Sex**								
Men	12.9	87.1	15.4	84.6	15.3	84.7	12.6	87.4
Women	21.8	78.2	22.7	77.3	20.4	79.6	17.3	82.7
**Age, yrs**								
16–29	20.7	79.3	26.6	73.4	18.9	81.1	14.8	85.2
30–49	18.1	81.9	20.8	79.2	14.6	85.4	10.1	89.9
50–64	15.3	84.7	16.1	83.9	17.1	82.9	13.8	86.2
65–85	17.3	82.7	17.0	83.0	21.2	78.8	21.0	79.0
**Education**								
Primary	14.7	85.3	15.0	85.0	19.6	80.4	19.9	80.1
Upper secondary	16.1	83.9	16.4	83.6	17.0	83.0	14.0	86.0
Post-secondary < 3 yrs	20.4	79.6	21.9	78.1	19.5	80.5	16.5	83.5
Post-secondary > 3yrs	18.3	81.7	21.9	78.1	16.7	83.3	12.7	87.3
**Living area**								
Major city^a^	17.8	82.2	21.1	78.9	18.0	82.0	14.1	85.9
City	17.5	82.5	20.3	79.7	19.1	80.9	15.7	84.3
Town	19.2	80.8	19.6	80.4	19.2	80.8	17.8	82.2
Rural area	15.6	84.4	14.4	85.6	13.0	87.0	12.4	87.6
**Total household income**								
Low	17.6	82.4	17.4	82.6	24.1	75.9	22.0	78.0
Middle	17.8	82.2	19.8	80.2	18.0	82.0	15.2	84.8
High	17.6	82.4	20.7	79.3	13.6	86.4	10.1	89.9
**Self-reported health**								
Poor	15.5	84.5	15.5	84.5	25.3	74.7	27.4	72.6
Fairly good	16.7	83.3	16.8	83.2	18.4	81.6	17.6	82.4
Good	16.8	83.2	19.1	80.9	17.1	82.9	13.6	86.4
Very good	19.5	80.5	21.7	78.3	16.6	83.4	12.7	87.3
**PHCC visits last year**								
None	14.9	85.1	17.5	82.5	12.6	87.4	9.1	90.9
1 or 2	18.4	81.6	19.6	80.4	15.8	84.2	12.2	87.8
3 or 4	15.0	85.0	17.1	82.9	19.2	80.8	17.2	82.8
5 or more	21.4	78.6	23.0	77.0	27.9	72.1	26.7	73.3
**Need to change lifestyle**^**b**^								
Smoking habits	25.9	74.1	-	-	-	-	-	-
Alcohol consumption	-	-	16.4	83.6	-	-	-	-
Eating habits	-	-	-	-	18.3	81.7	-	-
Physical activity	-	-	-	-	-	-	16.5	83.5

^a^Stockholm, Gothenburg, Malmö

^b^Participants who answered “yes” to the question about the “need to change lifestyles regarding …”. PHCC = Primary health care centre. Values are given in %. Likely refers to the response alternatives “very likely” and “fairly likely”, not likely refers to the response alternatives “not likely” and “not at all likely”

The likelihood of seeking support regarding lifestyle counselling from primary health care was significantly higher among the group with a perceived need versus no perceived need, for smoking habits (*P* = 0.008) and physical activity (*P* = 0.044). This was not the case however for alcohol consumption (*P* = 0.284) and eating habits (*P* = 0.704).

**Fig. 3:**
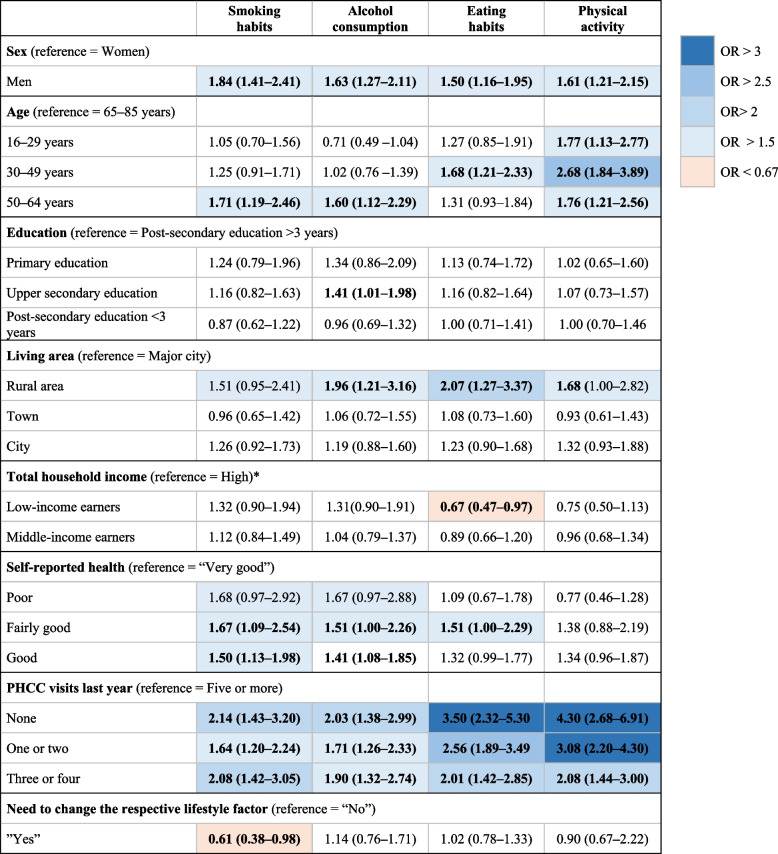
Logistic regression analysis for predicting who would be “not likely” to contact a primary health care centre regarding support for lifestyle changes, giving odds ratios (ORs) and 95% confidence intervals (CIs). Bold letters indicate significant ORs (*p* < 0.05). * “High” total household income: > 700 000 SEK per year. PHCC = primary health care centre

The logistic regression analysis presented in Fig. 3 showed that male sex predicted lower likelihood of contacting a primary health care centre, irrespective of the lifestyles investigated (OR range: 1.50–1.84). With living in a major city as reference, living in a rural area was a significant predictor of being less likely to contact a primary health care centre regarding alcohol consumption, eating habits, and physical activity (OR: 1.96, 2.07, and 1.68, respectively). Compared with high consumption of primary health care (five or more visits per year), lower consumption predicted lower likelihood of contacting a primary health care centre for all of the lifestyles investigated (OR range: 1.64–4.30), and this finding was particularly strong for both eating habits and physical activity (OR 2.01–4.30). Perceived need to change lifestyle was a predictor for only smoking habits and not the other lifestyles.

## Discussion

Our main finding that only a minority of the participants considered seeking support from a primary health care centre for lifestyle changes does not correspond to the overall trust in health care services in Sweden. In a survey from 2020, 69% of respondents reported that they had high or very high trust in healthcare in general. Regarding primary health care centres, 66% reported high or very high trust, but the trust in care at hospitals was even higher, with 76% of respondents reporting high or very high trust [25]. Two studies from Sweden found that trust in health care professionals made it easier to change lifestyles [26, 27]. Additionally, a population-based survey from 2016 showed that 97% of the respondents were positive to discuss their lifestyles with health care professionals in order to receive adequate care and treatment [28]. In our study, smoking habits and alcohol consumption were the lifestyles that were ranked as most likely to seek support from a primary health care centre for, and particularly evident in persons reporting a need to change smoking habits. This could be interpreted as meaning that people consider these lifestyles as more of risk factors to cause long-term ill health, and therefore important to seek support for and discuss with health care professionals.

Few yearly visits to a primary health care centre predicted a lower likelihood of seeking support from a primary health care centre for lifestyle changes. Reasons for this finding might include low visibility of primary health care centres in the community together with lack of awareness that these centres offer lifestyle counselling. Another possible reason could be that members of the general population contacts other actors in society for support regarding this issue. For example, they might contact a personal trainer or a health coach, or join a slimming club. Feng et al. [12] highlight alternative providers of lifestyle counselling for support in lifestyle changes. Altogether, it is likely that healthy lifestyles need to be approached from several angles in society, to help members of the general population to adopt healthier lifestyles. Earlier studies have shown that men are less likely to seek care [29, 30], and our study extends this finding by showing that it also applies to seeking lifestyle support in a primary health care setting. Novak et al. [31] suggest that men’s overall avoidance of health care might be related to their own perceptions of the male gender role as being tough and being able to push through pain, and that men’s health-seeking behaviour is affected by their perceptions of how helpful or knowledgeable a physician is. However, a Swedish study found that men were reported to be more likely than women to be asked about lifestyle issues in encounters with health care [8]. Further research is needed to explore gender disparities in health-seeking behaviour with regard to lifestyle counselling. Furthermore, we also found that living in a rural area predicted a lower likelihood of seeking support for lifestyle changes. The reason for this finding might be connected to accessibility in primary health care, which has been found to be essential in relation to patients’ needs of support in care [32]. A recent study from Sweden reported that rural areas and smaller cities have higher proportion of obese people in comparison to large cities [33], which suggests that accessibility of health care might play some role. The rising use of digital technology in society and health care implies a need to explore digital tools in lifestyle counselling, which may be more relevant for persons living in rural areas. A recent systematic review reported that digital self-monitoring behavioural interventions regarding physical activity and eating habits were effective in regard to supporting persons in weight loss, in comparison to interventions excluding digital self-monitoring [34]. Chatterjee et al. [35] found that successful digital interventions to promote healthier lifestyles was built upon several factors such as participants holding digital literacy and personalised feedback.

Although persons in our study perceived a need to change their lifestyles, few persons reported that they would be likely to seek such support from a primary health care centre. This finding brings the reasoning to stigmatization which is a well-known barrier, for example regarding smoking habits in which smokers are less likely than non-smokers to seek primary health care [36]. This raises the question of whether primary health care centres are able to reach the persons who are in most need of changing their lifestyle in this respect [12]. There is a fine and time-consuming balance between removing the blame associated with smoking and encouraging persons to actively seek help when needed [37]. In addition, obesity is a characteristic that is often stigmatized in relation to unhealthy lifestyles in which physicians and nurses are known to hold negative attitudes about obese persons [38]. Research indicates that the attitudes from health care providers can cause feelings of disrespect or of not being welcomed in obese patients, thus negatively affecting both the specific encounter and the individual’s willingness to seek care [39]. The impact of stigma in health care may explain the low likelihood of seeking lifestyle counselling from primary health care even among respondents who reported a perceived need to change their lifestyles. For this reason, attention needs to be given to acknowledging stigmatization as a barrier, in order both to support lifestyle changes and to reach those in most need. However, from a broader societal perspective, healthy diet and regular physical activity should be seen as valuable factors for health and well-being for all members in society, not only for persons with obesity.

In Sweden, structural efforts for increased health-promotive and disease-preventive work have been discussed, for example better routines in practice for lifestyle counselling along with an equality perspective for such tasks across the country, i.e. persons that seek support from a primary health care centre should be able to receive support to change lifestyles to the same extent regardless of which county council they live in [40]. A Swedish report from 2013 (two years after the implementation of the national clinical guidelines for unhealthy lifestyles), showed that reimbursement to caregivers can be roughly divided into three different categories: fixed, variable and special economic compensation. Fixed compensation refers to economic compensation within the framework of the basic mission of offering health-promotive and disease-preventive services for listed patients, for example annual compensation of 100 SEK per listed resident. Variable compensation is linked to specific clinical tasks that aims to change lifestyles, such as supporting patients to change eating habits, or prescribing physical activity on prescription. Also, variable economic compensation can be given when health care professionals have identified risk factors in patients, for example high blood pressure. Finally, some county councils can seek special economic compensation with the aim to develop the health-promotive and disease-preventive work, along with establishment of collaborations with other actors in society, to take greater responsibility for health preventive services in the surrounding area [41]. It is also important to highlight the individual expenses associated with a lifestyle counselling as the costs might be much higher if a person has initiated the lifestyle counselling themselves rather than if a health care professional have taken the initiative.

### Limitations and strengths

Some limitations of our study need to be addressed. There were no significant differences in geographical composition of respondents compared to the general population as a whole [42]. But notably age distribution and education level differed from the general population as the age group 16–29 years were underrepresented and persons with high level of education were overrepresented. Another limitation was that the questions were not validity and/or reliability tested before the study was conducted, which can be seen as a limitation. Furthermore, we had no data from the participants regarding outcome measures such as weight or body mass index, and no information about their current lifestyles, in terms of, for example eating habits or smoking; this prohibited us from conducting more in-depth analyses. Further, the data collection took place during the COVID-19 pandemic, which may have influenced the response rate and the answers given. However, the response rate of approximately 50% could be seen as a strength, as this is relatively high both for a postal survey and in comparison with international surveys [24]. Our study was conducted within the Swedish primary health care context, and so our findings may have limited generalizability to other countries due to differences between health care systems.

## Conclusion

This study provides knowledge about the likelihood of members of the general community contacting a primary health care centre regarding support for lifestyle changes. We can conclude that primary health care centres are not the main choice for lifestyle counselling, and we have identified factors predicting low likelihood of using this support. In light of the growing need for lifestyle changes, primary health care centres need to find valid methods for engaging with and meeting the needs of a population struggling with unhealthy lifestyles.

## Data Availability

The dataset supporting the conclusions of this article is available in the National SOM survey 2020, SOM Institute at University of Gothenburg, Version 1. https://doi.org/10.5878/jyym-aq24

## Abbreviations

NCDs: Non-communicable diseases
OR: Odds ratio
